# Phytochemical analysis and anticholinesterase activity of aril of *Myristica fragrans* Houtt

**DOI:** 10.1186/s13065-022-00897-9

**Published:** 2022-11-28

**Authors:** Arezoo Rastegari, Azadeh Manayi, Mahdi Rezakazemi, Mahdieh Eftekhari, Mahnaz Khanavi, Tahmineh Akbarzadeh, Mina Saeedi

**Affiliations:** 1 Persian Medicine and Pharmacy Research Center, Tehran University of Medical Sciences, Tehran, Iran; 2 Medicinal Plants Research Center, Faculty of Pharmacy, Tehran University of Medical Sciences, Tehran, Iran; 3 Department of Pharmacognosy, Faculty of Pharmacy, Tehran University of Medical Sciences, Tehran, Iran; 4 Department of Pharmacognosy and Pharmaceutical Biotechnology, Faculty of Pharmacy, Kermanshah University of Medical Sciences, Kermanshah, Iran; 5 Department of Medicinal Chemistry, Faculty of Pharmacy, Tehran University of Medical Sciences, Tehran, Iran

**Keywords:** AChE, BChE, Mace, Metal chelating, *Myristica fragrans* Houtt., Neuroprotectivity, Phytochemistry

## Abstract

In this study, the ethyl acetate fraction of *Myristica fragrans* Houtt. was investigated for its in vitro anticholinesterase activity as well as neuroprotectivity against H_2_O_2_-induced cell death in PC12 neuronal cells and the ability to chelate bio-metals (Zn^2+^, Fe^2+^, and Cu^2+^). The fraction was inactive toward acetylcholinesterase (AChE); however, it inhibited the butyrylcholinesterase (BChE) with IC_50_ value of 68.16 µg/mL, compared with donepezil as the reference drug (IC_50_ = 1.97 µg/mL) via Ellman’s method. It also showed good percentage of neuroprotection (86.28% at 100 µg/mL) against H_2_O_2_-induced neurotoxicity and moderate metal chelating ability toward Zn^2+^, Fe^2+^, and Cu^2+^. The phytochemical study led to isolation and identification of malabaricone A (**1**), malabaricone C (**2**), 4-(4-(3,4-dimethoxyphenyl)-2,3-dimethylbutyl)benzene-1,2-diol (**3**), nectandrin B (**4**), macelignan (**5**), and 4-(4-(benzo[*d*][1,3]dioxol-5-yl)-1-methoxy-2,3-dimethylbutyl)-2-methoxyphenol (**6**) which were assayed for their cholinesterase (ChE) inhibitory activity. Compounds **1** and **3** were not previously reported for *M. fragrans*. Among isolated compounds, compound **2** showed the best activity toward both AChE and BChE with IC_50_ values of 25.02 and 22.36 μM, respectively, compared with donepezil (0.07 and 4.73 μM, respectively).

**Supplementary Information:**

The online version contains supplementary material available at 10.1186/s13065-022-00897-9.

## Introduction

Alzheimer’s disease (AD) is a progressive neurodegenerative disorder, characterized as the main cause of dementia among older adults. It has become a serious health concern as approximately 50 million individuals suffer from AD worldwide, ranking it the fifth leading cause of death in the world. The prevalence of AD is expected to increase to 68% in low- and middle-income countries by 2050 [1]. Despite the budget and efforts specified to the management of AD, there is no certain cure because it is a multi-factorial disorder as multiple underlying mechanisms are involved in the pathogenesis of disease [2]. Although the origin of AD is still unclear, clinical diagnosis and autopsy studies have identified important neuropathological hallmarks responsible for the onset and progression of the disease. Aggregation and extracellular deposition of amyloid beta (Aβ) plaques, that is usually catalyzed by β-secretase 1 (BACE-1), leads to the activation of neuron death. Another mechanism is the intracellular formation of neurofibrillary tangles (NFTs) and neuropil threads (NTs), caused by the abnormal hyperphosphorylation of tau protein. Also, the disruption of metal-ion regulation has been found to interact with the Aβ, enhancing the aggregation and formation of plaques. Another important pathological pathway, known as cholinergic hypothesis, is related to the reduction of acetylcholine (ACh) levels by cholinesterases (ChEs) in the brain [3]. In this respect, cholinesterase inhibitors (ChEIs) have played an essential role in reducing the symptoms and possibly slowing the rate of progression of the disease [4].

It has been depicted that single-target drug therapies have not offered an efficient therapeutic strategy for the treatment of AD and therefore, discovery of multi-target agents has been in the center of attention in the field of drug development research [5]. A variety of medicinal plants have been vastly applied for memory enhancement and other dementia-related complications via various mechanisms [6] such as ChE inhibitory activity [7–10], as well as prevention of neurodegenerative diseases [11].

*Myristica fragrans* Houtt., belonging to the family Myristicaceae, known as nutmeg tree, is a tropical evergreen and aromatic tree possessing a pleasant aroma and taste. Nutmeg is the dried kernel of the ripe seed and mace is the red lacy layer (aril) surrounding the seed. It has been widely used as spices as well as remedies for various disease in folk and modern medicine. A wide range of phytochemicals including lignans, neolignans, diphenylalkanes, phenylpropanoids, terpenes, alkanes, fatty acid, and fatty acid esters, steroids, saponins, triterpenoids, flavonoids, and 2-alkylcyclobutanones have been identified in *M. fragrans* [12] (Additional file 1: Table S1).

Recently, we have investigated some biological activities of the ethyl acetate fraction of *M. fragrans* seeds related to AD. It selectively inhibited the butyrylcholinesterase (BChE) and showed no activity toward acetylcholinesterase (AChE) [13]. In this study, we focused on the aqueous extract as well as *n*-hexane, chloroform, and ethyl acetate fractions of the methanol extract of the mace (the aril of *M. fragrans*) to evaluate their anti-ChE activity. As the ethyl acetate showed the best activity, it was candidate for the investigation of its neuroprotectivity and metal chelating ability as well as phytochemical analysis.

## Experimental

### Plant

The aril of *M. fragrans* was purchased from Tehran market, Iran. It was identified and deposited in the herbarium of Faculty of Pharmacy, Tehran University of Medical Sciences, Tehran, Iran, with the voucher number of PMP-1620. It is confirmed that all methods were performed in accordance with the relevant guidelines and regulations.

### Extraction

The dried aril of *M. fragrans* was milled using a laboratory-scale mill and then the powder was extracted as described below:

### Aqueous extract

It was obtained by boiling powdered plant (50 g) in distilled water (750 mL) in a beaker for 10 min. Then, it was cooled, filtered off, and the solid residue was re-extracted by 250 mL distilled water. Finally, the extract was filtered off, centrifuged at 4000 rpm for 6 min, concentrated using a rotary evaporator under vacuum at 50 °C (Heidolph, Heizbad Hei-VAP, Germany), and freeze-dried (LTE science LTD, England) at -60 °C/10 μmHg for 8 h to give the aqueous extract in 20.04% yield. It was stored at − 20 °C.

### Hydroalcoholic extract

It was prepared by the maceration of the powdered plant (1900 g) in methanol–water (80:20 (v/v)) with total volume of 1500 mL for 72 h at room temperature. The extraction was repeated three times and finally the collected extract was filtered off, centrifuged at 4000 rpm for 6 min (Heraeus Megafuge 1.0, England), concentrated using a rotary evaporator under vacuum at 40 °C (Heidolph, Heizbad Hei-VAP, Germany), and freeze-dried (LTE science LTD, England) at − 60 °C/10 μmHg for 8 h to obtain desired extract in 20.89% yield. It was stored at − 20 °C.

### Liquid–liquid fractionation

Dry methanolic extract (400 g) was dissolved in methanol-distilled water (500 mL, 80:20 (v/v)) and the solution was then subsequently fractionated by a series of liquid–liquid extractions using *n*-hexane (three times, totally 3000 mL), chloroform (three times, totally 3000 mL), and ethyl acetate (three times, totally 3000 mL). All fractions were concentrated using a rotary evaporator under vacuum at room temperature and freeze-dried (LTE science LTD, England) at − 60 °C/10 μmHg for 8 h to afford related fractions in 19.18, 15.97, and 25.42% yield, respectively.

### Isolation of compounds

The ethyl acetate fraction (5 g) was loaded onto a silica gel column (Merck 230–400 mesh), eluted with a gradient mixture of EtOAc/*n*-hexane (30:70 to 100:0), and five  sub-fractions (A1-5) were collected. A3 (800 mg) was loaded onto a Sephadex® LH-20 and eluted with methanol to obtain eight sub-fractions (B1-8). Also, A2 (450 mg) was loaded onto a Sephadex^®^ LH-20 and eluted with methanol to afford two sub-fractions (C1 and C2).

Further purification was performed on B5 (160 mg) which was subjected to column chromatography on silica gel (Merck 230–400 mesh) and eluted with a gradient mixture of EtOAc/*n*-hexane (5:95 to 50:50) leading to the isolation of two compounds **1** (77 mg), and **2** (83 mg). Purification of B3 (220 mg) using a column of silica gel (Merck 230–400 mesh) and elution with a gradient mixture of EtOAc/*n*-hexane (5:95 to 50:50) gave five sub-fractions (D1-5). Among them, D1 (93 mg) and D4 (123 mg) were pure compounds, known as compounds **3** and **4**. Furthermore, C2 (330 mg) was subjected to column chromatography on silica gel (Merck 230–400 mesh) and eluted with a gradient mixture of EtOAc/*n*-hexane (5:95 to 30:70) to afford three compounds, **5** (128 mg), **6** (66 mg), and **1** (118 mg).

### *In vitro *ChE inhibitory activity

Acetylcholinesterase (AChE, E.C. 3.1.1.7, Type V-S, lyophilized powder, from electric eel, 1000 unit), butyrylcholinesterase (BChE, E.C. 3.1.1.8, from equine serum), acetylthiocholine iodide (ATCI), butyrylthiocholine iodide (BTCI), and 5,5-dithiobis-(2-nitrobenzoic acid) (DTNB) were purchased from Sigma-Aldrich. Potassium dihydrogen phosphate, dipotassium hydrogen phosphate, potassium hydroxide, and sodium hydrogen carbonate were obtained from Fluka.

The in vitro cholinesterase inhibitory activity of the aqueous extract, fractions, and isolated compounds was studied using the modified Ellman's method, exactly according to our previous study [14].

To obtain acceptable enzyme inhibitory activity (20–80%), the stock solutions of the fractions (10 mg/mL) and compounds (1 mg/mL) were prepared in DMSO and were diluted with a mixture of DMSO and methanol to achieve four different final concentrations of the fractions (63.5, 125, 250, 500 μg/mL) and compounds (1, 10, 20, 40 μg/mL), while obtaining the final ratio of 50/50 DMSO/methanol. Each well consisted of 50 μL potassium phosphate buffer (KH_2_PO_4_/ K_2_HPO_4_, 0.1 M, pH 8), 25 μL of the prepared sample as described above and AChE (25 μL) with final concentration of 0.22 Units/mL in buffer. They were pre-incubated for 15 min at room temperature and then 125 μL of DTNB (3 mM in buffer) was added to the mixture. Changes in the absorbance were measured spectrometrically at 405 nm, followed by the addition of 25 μL of the substrate (ATCI, 3 mM in water).

In parallel, a blank containing all components without enzyme was used in order to account the non-enzymatic reaction. A negative control was also performed under the same conditions without inhibitor, and donepezil was used as the positive control. The IC_50_ values were determined graphically from log concentration vs. inhibition (%) curves. All experiments were performed in triplicate. BChE inhibition assay was also performed in the same method using BTCI as the substrate.

### Kinetic studies

Kinetic studies of compound **2** were performed for the inhibition of ChEs based on the Ellman's method, using various concentrations of the inhibitor [14]. In the case of inhibition of AChE, the inhibitor was used at the concentrations of 0, 28, 56, and 112 μM and in the case of inhibition of BChE, it was used as 0, 14, 28, and 56 μM. The Lineweaver–Burk reciprocal plot was constructed by plotting 1/V against 1/[S] at variable concentrations of the substrate, ATCI (187.5, 750, 1500, 3000 µM) or BTCI (187.5, 750, 1500, 3000 µM).

### Neuroprotectivity against H_2_O_2_-induced neurotoxicity in PC12 cells

PC12 cell line was obtained from Pasteur institute and all culture media as well as supplements were purchased from Gibco. The cells were cultivated in DMEM supplemented with 10% fetal calf serum plus antibiotics (100 units/mL penicillin, 100 μg/mL streptomycin). To induce neuronal differentiation, PC12 cells were re-suspended using trypsin/EDTA (0.25%) and seeded in 96 well culture plate (4000cells/well) and cultured for 1 week in differentiation medium (DMEM + 2% horse serum + NGF (100 ng/mL) + penicillin & streptomycin). The neuroprotection assay against H_2_O_2_-induced cell death in PC12 cells was exactly performed according to our previous report [13].

To investigate the effect of the fraction on the survival rate of neurons, the culture medium was changed to NGF free medium and different concentrations of the fraction (1, 10, 100 μg/mL) were applied on cells, compared with quercetin (50 µM) as the positive control. The fraction was diluted in DMEM and a volume of 10 μL was added to each well. Then, after 3 h, induction of ROS mediated apoptosis was initiated by adding H_2_O_2_ (400 μM) to their medium. After 12 h, MTT assay was performed. MTT solution (5 mg/mL) was added to each well in a volume of 10 μL, and 3.5 h later, 100 μL of the solubilisation solution (10% SDS in 0.01 M HCl (w/v)) was added into each well. The plates were allowed to stand overnight in the incubator in a humidified atmosphere. Absorbance was measured at 570 nm with a reference wavelength of 630 nm using a microplate reader (BioTek ELx808, USA). Each experiment was conducted in three replicates.

### Metal chelating ability

To investigate the biometal chelating ability of the ethyl acetate fraction of aril of *M. fragrans*, the absorbance of the methanolic solution was initially recorded at the concentration of 25 µg/mL in the wavelength range of 250–600 nm. Then, to study the chelating ability of the fraction toward metal ions (Zn^2+^, Fe^2+^, and Cu^2+^), an equal volume of the fraction solutions (final concentration of 25 µg/mL) and the desired metal ion (final concentration of 20 μM) were mixed and placed at room temperature for 30 min. Then, the absorbance of the solution was read in the wavelength range of 250–600 nm and the results were compared with that obtained from the fraction alone [15].

## Results and discussion

### *In vitro *ChE inhibitory activity

Anti-ChE activity of aqueous extract and different fractions of the methanol extract of the aril of *M*. *fragrans* was evaluated and compared with donepezil as the reference drug (Table 1). As shown in Table 1, the aqueous extract depicted no AChEI and BChEI activity. Also, all fractions of the methanol extract could not inhibit AChE. However, they were found to be moderate to good inhibitors of BChE and among them, the ethyl acetate fraction showed the best anti-BChE activity with IC_50_ value of 68.16 μg/mL.

**Table1:** Anti-ChE activity of the aqueous extract and different fractions of the aril of *M. fragrans*^a^

Samples	AChEI [IC_50_ (μg/mL)]	BChEI [IC_50_ (μg/mL)]
Aqueous extract	> 500	> 500
*n*-Hexane fraction	> 500	288.95 ± 0.35
Chloroform fraction	> 500	177.07 ± 0.71
Ethyl acetate fraction	> 500	68.16 ± 0.67
Donepezil	0.03 ± 0.00	1.97 ± 0.03

^a^Data are expressed as mean ± SD (three independent experiments)

### Neuroprotectivity of the ethyl acetate fraction of aril of *M. fragrans* against H_2_O_2_-induced cell death in PC12 cells

**Fig. 1:**
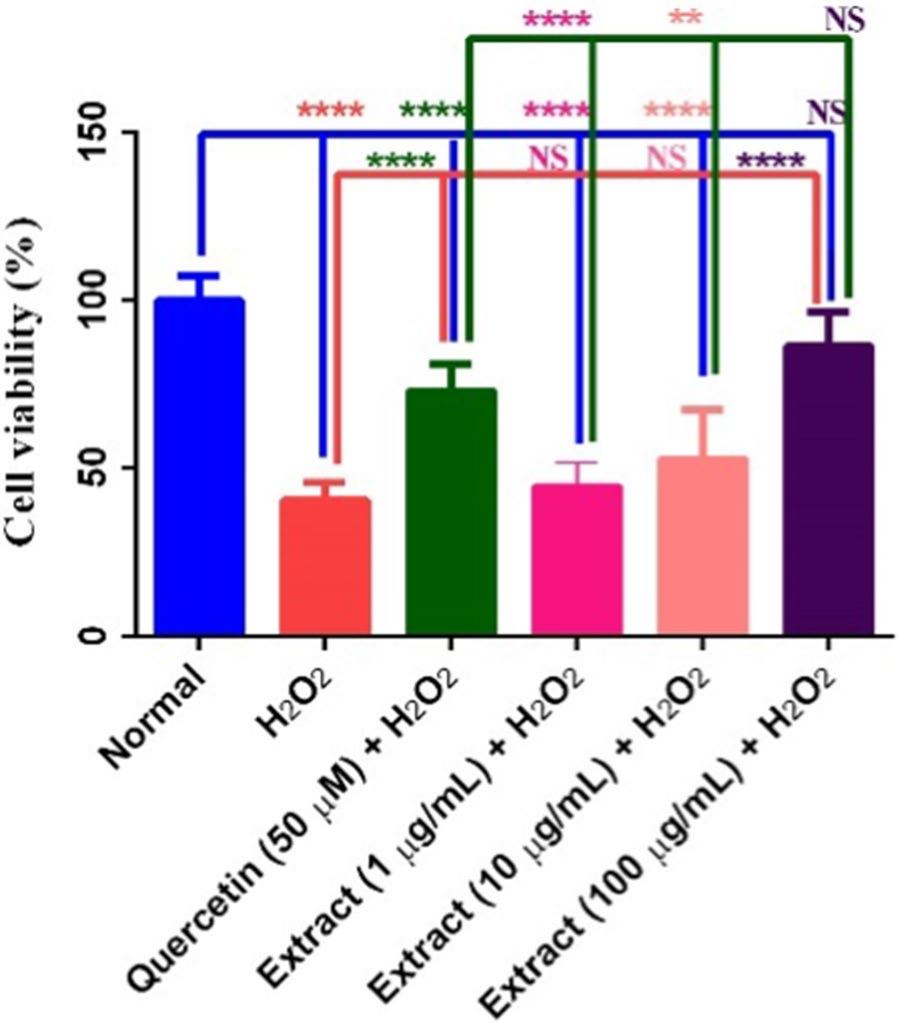
Neuroprotective effect of the ethyl acetate fraction of the aril of *M. fragrans* on survival of H_2_O_2_-treated neurons. Data are expressed as mean ± SD and one-way analysis of variance (ANOVA) followed by Tukey's multiple comparisons test was used to determine the level of significance (****P < 0.0001 and **P < 0.01)

Neuroprotective effect of the ethyl acetate fraction aril of *M. fragrans* at different concentrations of 1, 10, and 100 µg/mL was investigated against oxidative damage induced by H_2_O_2_ on PC12 cells, compared with the intact (normal, no intervention), quercetin + H_2_O_2_-treated (positive control), and H_2_O_2_-treated (negative control) cells (Fig. 1). It showed good neuroprotectivity at the above- mentioned concentrations by 44.38, 52.56, and 86.28%, respectively. It should be noted that the activity of the quercetin was recorded as 72.79% at 50 µM.

### Metal chelating ability of the ethyl acetate fraction of aril of *M. fragrans*

**Fig. 2:**
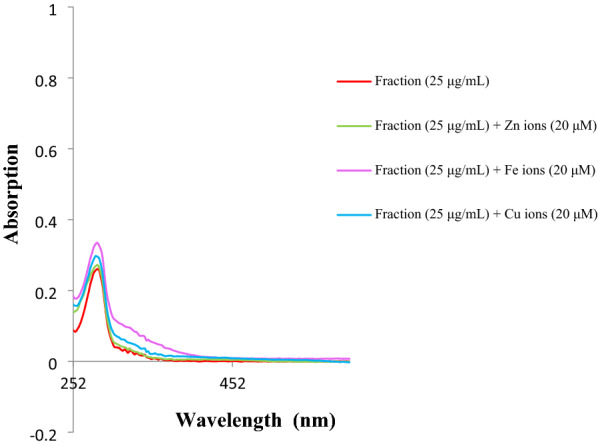
The absorbance changes of the ethyl acetate fraction of the mace alone and in the presence of Zn^2+^, Fe^2+^, and Cu^2+^ ions

To measure the metal chelating ability of the ethyl acetate fraction, the UV–visible absorption spectrum of the methanolic solution of the fraction was initially recorded in the range of 250–600 nm at the concentration of 25 μg/mL, showing an absorbance peak at 282 nm (Fig. 2). When the extract treated with the solutions of Zn^2+^, Fe^2+^, and Cu^2+^ ions (final concentration of 20 μM), no remarkable changes in the corresponding wavelengths (λ_max_) was observed. However, a slight blue-shift was ascribed to the interaction of the fraction with Zn^2+^ ions. In the case of Fe^2+^ and Cu^2+^ ions, the change of absorbance intensity was only observed.

### Isolation of compounds from the ethyl acetate fraction

**Fig. 3:**
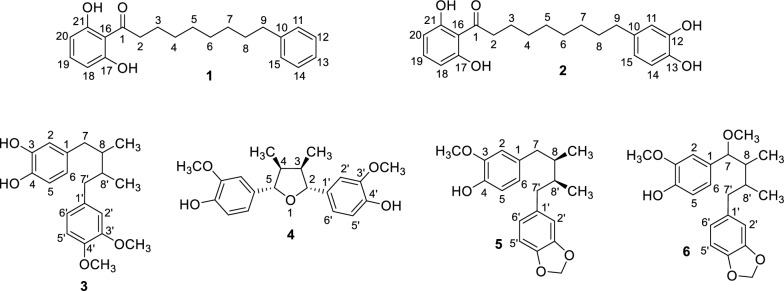
Isolated compounds from the ethyl acetate fraction of aril of *M. fragrans*

Phytochemical study of the ethyl acetate fraction of aril of *M. fragrans* led to the isolation of six compounds **1**–**6** (Fig. 3) as characterized below. The NMR spectra of **1**–**6** were compared with those reported in the literature [16–21]. It should be noted that compounds **1** and **3** have not been previously reported for *M. fragrans*.

#### Compound 1: 1-(2,6-Dihydroxyphenyl)-9-phenylnonan-1-one (Malabaricone A)

Mw = 326.19. ^1^H NMR (500 MHz, DMSO-*d*_*6*_): 7.20 (t, *J* = 8.3 Hz, 1H, H19), 6.94 (d, *J* = 7.9 Hz, 2H, H11, H15), 6.67–6.66 (m, 3H, H12, H13, H14), 6.37 (d, *J* = 8.3 Hz, 2H, H18, H20), 3.04 (t, *J* = 6.4 Hz, 2H, CH_2_-2), 2.43 (t, *J* = 7.5 Hz, 2H, CH_2_-9), 1.62–1.55 (m, 2H, CH_2_-3), 1.51–1.44 (m, 2H, CH_2_-8), 1.26–1.16 (m, 8H, CH_2_-4, 5, 6, 7). ^13^C NMR (125 MHz, DMSO-*d*_*6*_): 207.9 (C1), 161.6 (C17&21), 144.8 (C10), 135.7 (C19), 132.8 (C12&14), 129.4 (C11&15), 121.5 (C13), 115.4 (C16), 107.6 (C18&20), 44.5 (C2), 34.8 (C9), 31.8 (C8), 29.4 (C4), 29.3 (C5), 29.2 (C6), 29.1 (C7), 24.4 (C3).

Relation of coupling protons was determined by cross peaks in the ^1^H-^1^H COSY spectrum.

#### Compound 2: 1-(2,6-Dihydroxyphenyl)-9-(3,4-dihydroxyphenyl)nonan-1-one (Malabaricone C)

Mw = 358.18. ^1^H NMR (500 MHz, DMSO-*d*_*6*_): 7.20 (t, *J* = 8.1 Hz, 1H, H19), 6.63 (d, *J* = 8.0 Hz, 1H, H14), 6.57 (s, 1H, H11), 6.41 (d, *J* = 8.0 Hz, 1H, H15), 6.37 (d, *J* = 8.1 Hz, 2H, H18, H20), 3.04 (t, *J* = 7.4 Hz, 2H, CH_2_-2), 2.38 (t, *J* = 7.7 Hz, 2H, CH_2_-9), 1.60–1.56 (m, 2H, CH_2_-3), 1.50–1.44 (m, 2H, CH_2_-8), 1.30–1.16 (m, 8H, CH_2_-4, 5, 6, 7). ^13^C NMR (125 MHz, DMSO-*d*_*6*_): 207.9 (C1), 161.7 (C17&21), 145.4 (C12), 143.5 (C13), 133.8 (C19), 133.7 (C10), 119.3 (C15), 116.1 (C11), 115.8 (C14), 111.2 (C16), 107.6 (C18&20), 44.5 (C2), 35.0 (C9), 31.7 (C8), 29.5 (C4), 29.4 (C5), 29.3 (C6), 29.1 (C7), 24.4 (C3).

#### Compound 3: 4-(4-(3,4-Dimethoxyphenyl)-2,3-dimethylbutyl)benzene-1,2-diol

Mw = 330.18. ^1^H NMR (500 MHz, DMSO-*d*_*6*_): 8.64 (s, 2H, 2 × OH), 6.68–6.64 (m, 2H, H5', H6'), 6.65 (s, 1H, H2'), 6.61 (s, 1H, H2), 6.55 (d, *J* = 7.9 Hz, 1H, H5), 6.48 (d, *J* = 7.9 Hz, 1H, H6), 3.73 (s, 3H, OCH_3_), 3.70 (s, 3H, OCH_3_), 2.67 (dd, *J* = 13.4, 5.5 Hz, 1H, H7'a), 2.48 (dd, *J* = 13.7, 5.5 Hz, 1H, H7a), 2.30 (dd, *J* = 13.7, 8.5 Hz, 1H, H7b), 2.21 (dd, *J* = 13.4, 8.5 Hz, 1H, H7'b), 1.71–1.67 (m, 2H, H8, H8'), 0.79–0.76 (m, 6H, 2 × CH_3_). ^13^C NMR (125 MHz, DMSO-*d*_*6*_): 147.8 (C3'), 147.7 (C4'), 144.8 (C3), 144.2 (C4), 132.8 (C1), 132.5 (C1'), 121.5 (C6), 121.4 (C6'), 115.6 (C2), 115.5 (C5), 113.4 (C2'), 111.3 (C5'), 56.0 (OCH_3_), 55.9 (OCH_3_), 40.9 (C8), 39.0 (C8'), 38.5 (C7'), 37.7 (C7), 16.5 (CH_3_), 14.2 (CH_3_).

#### Compound 4: 4,4'-((2R,3R,4S,5S)-3,4-Dimethyltetrahydrofuran-2,5-diyl)bis(2-methoxyphenol) (Nectandrin B)

Mw = 344.16. ^1^H NMR (500 MHz, DMSO-*d*_*6*_): 8.90 (s, 2H, 2 × OH), 6.98 (s, 2H, 2 × H2'), 6.83 (d, *J* = 7.8 Hz, 2H, H5'), 6.78 (d, *J* = 7.8 Hz, 2H, H6'), 4.36 (d, *J* = 5.7 Hz, 2H, H2, H5), 3.78 (s, 6H, 2 × OCH_3_), 2.23–2.20 (m, 2H, H3, H4), 0.94 (d, *J* = 5.6 Hz, 6H, 2 × CH_3_). ^13^C NMR (125 MHz, DMSO-*d*_*6*_): 147.9 (C3'), 146.4 (C4'), 133.6 (C1'), 119.3 (C6'), 115.6 (C5'), 110.9 (C2'), 87.0 (C2&5), 55.9 (OCH_3_), 44.4 (C3&4), 13.1 (CH_3_).

#### Compound 5: 4-(4-(Benzo[*d*][1,3]dioxol-5-yl)-2,3-dimethylbutyl)-2-methoxyphenol (Macelignan)

Mw = 328.17. ^1^H NMR (500 MHz, DMSO-*d*_*6*_): 8.66 (s, 1H, OH), 6.80 (d, *J* = 7.9 Hz, 1H, H5'), 6.73 (s, 1H, H2'), 6.69–6.67 (m, 2H, H2, H6'), 6.63 (d, *J* = 8.0 Hz, 1H, H5), 6.54 (d, *J* = 8.0 Hz, 1H, H6), 5.96–5.95 (m, 2H, OCH_2_), 3.73 (s, 3H, OCH_3_), 2.70 (dd, *J* = 13.0, 5.0 Hz, 1H, H7'a), 2.66 (dd, *J* = 13.0, 5.0 Hz, 1H, H7'b), 2.24 (dd, *J* = 13.0, 9.2 Hz, 1H, H7a), 2.19 (dd, *J* = 13.0, 9.2 Hz, 1H, H7b), 1.68–1.64 (m, 2H, H8, H8'), 0.78–0.74 (m, 6H, 2 × CH_3_). ^13^C NMR (125 MHz, DMSO-*d*_*6*_): 147.8 (C3'), 147.6 (C3), 145.6 (C4'), 144.8 (C4), 135.8 (C1), 132.7 (C1'), 122.1, 121.5, 115.6, 113.3, 109.6, 108.3, 101.0, 56.5 (OCH_3_), 39.2 (C8), 39.1 (C8'), 38.6 (C7'), 38.4 (C7), 16.5 (CH_3_), 16.4 (CH_3_).

#### Compound 6: 4-(4-(Benzo[*d*][1,3]dioxol-5-yl)-1-methoxy-2,3-dimethylbutyl)-2-methoxyphenol

Mw = 358.18. ^1^H NMR (500 MHz, DMSO-*d*_*6*_): 6.96 (d, *J* = Hz, 1H, H5), 6.89–6.87 (m, 2H, H2, H6), 6.70–6.66 (m, 2H, H2', H5'), 6.55 (d, *J* = 7.9 Hz, 1H, H6'), 6.01–6.00 (m, 2H, OCH_2_), 4.36 (d, *J* = 9.8 Hz, 1H, H7), 3.75 (s, 3H, OCH_3_), 3.72 (s, 3H, OCH_3_), 2.66 (dd, *J* = 13.3, 5.0 Hz, 1H, H7'a), 2.44–2.31 (m, 1H, H8), 2.20 (dd, *J* = 13.3, 9.5 Hz, 1H, H7'b), 1.72–1.63 (m, 1H, H8'), 0.91 (d, *J* = 6.7 Hz, 3H, CH_3_), 0.78 (d, *J* = 6.7 Hz, 3H, CH_3_). ^13^C NMR (125 MHz, DMSO-*d*_*6*_): 147.6 (C3'), 147.4 (C3), 146.1 (C4'), 145.7 (C4), 137.6 (C1), 133.5 (C1'), 123.0 (C6'), 121.1 (C6), 114.3 (C5), 109.8 (C2), 108.7 (C2'), 108.4 (C5'), 100.7, 89.6 (C7), 55.8 (OCH_3_), 54.8 (OCH_3_), 46.3 (C8), 37.6 (C8'), 35.6 (C7'), 20.6 (CH_3_), 11.8 (CH_3_).

### In vitro ChE inhibitory activity of isolated compounds

Anti-ChE activity of the compounds **1**–**6** was assessed against AChE and BChE, compared with donepezil as the reference drug (Table 2).

**Table 2:** Anti-ChE activity of the isolated compounds from the aril of *M. fragrans*^a^

Compound	AChEI (μM)	BChEI (μM)
**1**	67.41 ± 1.52	27.16 ± 0.06
**2**	25.02 ± 0.95	22.36 ± 0.03
**3**	(0% at 40 µg/mL)	(47.35% at 40 µg/mL)
**4**	(0% at 40 µg/mL)	(45.84% at 40 µg/mL)
**5**	(12.8% at 40 µg/mL)	(54.41% at 40 µg/mL)
**6**	(0% at 40 µg/mL)	(44.05% at 40 µg/mL)
Donepezil	0.07 ± 0.00	4.73 ± 0.91

^a^Data are expressed as mean ± SD (three independent experiments)

As can be seen in Table 2, compounds **1** and **2** were potent inhibitors of both ChEs. Although the ethyl acetate fraction was not active toward AChE, compounds **1** and **2** depicted desired inhibitory activity.

### Kinetic studies

**Fig. 4:**
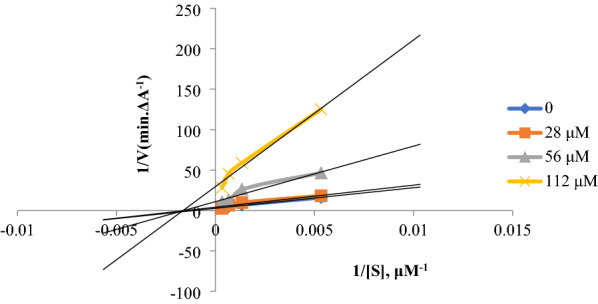
Kinetic study of compound **2** against AChE. Lineweaver–Burk plot and double reciprocal Lineweaver–Burk plot are shown

**Fig. 5:**
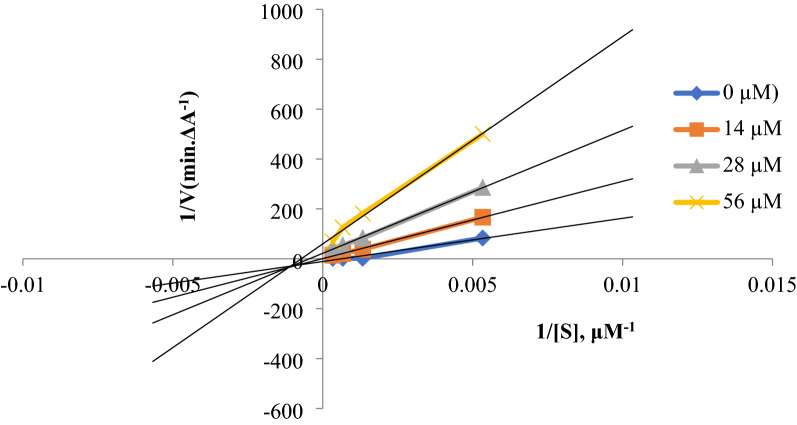
Kinetic study of compound **2** against BChE. Lineweaver–Burk plot and double reciprocal Lineweaver–Burk plot are shown

Kinetic studies were performed to investigate the mechanism of inhibition by the most potent inhibitor (compound **2**) against AChE and BChE (Fig. 4 and Fig. 5). Graphical analysis of the reciprocal Lineweaver–Burk plots demonstrated a non-competitive inhibition toward both enzymes, indicating that compound **2** can bind to the both CAS and PAS of the ChEs. In addition, the Ki values for the inhibition of AChE and BChE were calculated as 25.01 and 22.36 μM, respectively.

AD is a complex disorder and various aspects on the pathogenesis of the disease have remained unclear in such a manner that memory and cognitive decline and behavioral disorders are still serious complications. It is believed that improvement of cholinergic transmission leads to the cholinergic receptors stimulation or increasing the availability of ACh in the synaptic cleft and consequently, alleviation of symptoms of AD. Also, the role of abnormal cholinergic system in the promotion of amyloid precursor protein (APP) metabolism and tau phosphorylation which lead to neurotoxicity, neuroinflammation and neuronal death, has been proven [22]. Thus, inhibition of AChE which catalyzes the transfer of an acetyl group from acetyl-CoA to choline, can be considered as a strong tool. BChE is a closely related enzyme known as a pseudo cholinesterase, catalyzes the breakdown of different choline esters. The inhibition of BChE is also useful as it bears 65% of structural resemblance with AChE [23].

Many previous studies have indicated anti-ChE activity of the medicinal plants as an effective tool in the treatment of AD [24]. In this respect, galantamine and Huperzine A are approved as natural-based anti-AD products which significantly help to improve cognitive symptoms through the inhibition of AChE [25]. Also, *Ginkgo biloba* has been known as the versatile herbal medicine depicting good results in the inhibition of AChE as well as preclinical and clinical trials [26, 27]. Moreover, plants are usually rich in antioxidant phenolic compounds, possessing multi-target neuroprotective agents against AD [28].

 Considering the efficacy of medicinal plants, we focused on *Myristica fragrans* Houtt., both its seeds [13] and aril, which has been used for the memory improvement in Persian medicine. Similarly, aqueous extract of both seeds and aril showed no ChE activity. All fractions of the methanol extract were inactive toward AChE, however, they were BChE inhibitors. In both studies, the ethyl acetate fraction showed the best BChEI activity. Comparing our results with those obtained from *M*. *fragrans* seeds [13] revealed similar trend in the ChEI activity of the fractions, however, the ethyl acetate fraction of the aril (IC_50_ = 68.16 µg/mL) demonstrated higher anti-BChE activity than that of seeds (IC_50_ = 145.84 μg/mL). The selective inhibition of the BChE can be achieved by the bulky inhibitors due to the slight difference in the structure of the deep gorge with that of AChE [23]. In this respect, the selective inhibition of BChE by different fractions of the plant can be explained. Selective inhibition of BChE would be appropriate for the treatment of mid- to severe AD patients. However, after isolation of compounds **1**–**6** from the ethyl acetate fraction, they were evaluated for their both AChE and BChE. Although compounds **3**–**6** were weak inhibitors of both enzymes, compounds **1** and **2** were strong inhibitors. Compound **2** (IC_50_ = 25.02 and 22.36 μM against AChE and BChE, respectively) was especially more potent than compound **1** (IC_50_ = 67.41 and 27.16 μM against AChE and BChE, respectively) which non-competitively inhibited both enzymes, according to the kinetic studies.

It is clear that the death of neurons is a significant feature of the neurodegenerative diseases such as AD. It is definitely proven that high production of free radicals via elevated oxidative cellular stress in the brain, is the main cause of AD. The oxidative stress occurs by the reduction of polyunsaturated fatty acid, increase of protein and DNA oxidation and lipid peroxidation as well as the aggregation and accumulation of Aβ [28]. Based on the results from phytochemical analysis, the ethyl acetate fraction is enriched in lignans and phenolic compounds. The protectivity of these compounds against neuronal injury and neurodegradation has been fully discussed in the literature. The neuroprotectivity of phenolic compounds is generally fulfilled through the inhibition of ChEs [29]. In this regard, desired neuroprotectivity of the ethyl acetate fraction of aril of *M. fragrans* against H_2_O_2_-induced cell death in PC12 neurons can be explained. Comparing the neuroprotectivity of the ethyl acetate fraction of the aril with that of seeds [13] indicated higher activity of seeds (55.1, 88.6, and 93.3% at the same concentrations, respectively).

The relation between redox-active metal ions (e.g. Zn^2+^, Fe^2+^, and Cu^2+^) and AD is also characterized by their role in inducing oxidative stress and misfolding and aggregation of Aβ. They stimulate oxidative reactions in living organisms by lowering their activation energy to produce harmful reactive oxygen species. Aβ catalyzes the reduction of bio-metals and the reduced forms react with hydrogen peroxide (H_2_O_2_) as an intercellular signaling molecule and neuromodulator in the brain, affording radicals that damage DNA, lipid peroxidation, and alteration of mitochondrial membrane potential. This is a complex pathway and metal chelating agents that can remedy abnormal Aβ–metal interactions are in the center of attention [30]. Results from metal chelating ability of the ethyl acetate fraction revealed moderate activity, however, it was more potent toward Zn^2+^ ions. It has been perceived that zinc plays numerous functions in the brain, both in health and in diseases such as AD. It is essential in the enzymatic nonamyloidogenic processing of the APP and in the enzymatic degradation of the Aβ peptide. Zinc binds to Aβ to form neurotoxic species resulting in synaptic and memory deficits. Thus, it is clear that chelation of zinc ions can be a potential therapeutic approach [31].

## Conclusion

Our study was conducted based on our previous report on the anti-AD activity of *M. fragrans* seeds and emphasis of Persian medicine on the memory enhancing properties of the plant. The ethyl acetate fraction of mace showed the best and selective BChE inhibitory activity (IC_50_ = 68.2 μg/mL). This fraction also demonstrated high neuroprotectivity against H_2_O_2_-induced cell death on PC12 neurons (86.3% at 100 μg/mL). However, moderate metal chelating ability toward Zn^2+^, Fe^2+^, and Cu^2+^ ions was afforded. The phytochemical analysis of the ethyl acetate fraction gave six compounds and among them, malabaricone C (**2**) showed the best activity against both enzymes (IC_50_ = 22.05 and 22.36 μM on AChE and BChE, respectively).

## Supplementary Information

**Additional file 1: Table S1.** Isolated compounds from the aril of *M. fragrans.*

## Data Availability

All data generated or analyzed during this study are included in this published article and its Additional information files.

## Abbreviations

ACh: Acetylcholine
AChE: Acetylcholinesterase
Aβ: Amyloid beta
AD: Alzheimer’s disease
APP: Amyloid precursor protein
ATCI: Acetylthiocholine iodide
BACE-1: β-Secretase
BChE: Butyrylcholinesterase
BTCI: Butyrylthiocholine iodide
ChE: Cholinesterase
DTNB: 5,5-Dithiobis-(2-nitrobenzoic acid)
mL: Milliliter
μg: Microgram
MTT: (3-[4,5-Dimethylthiazol-2-yl]-2,5 diphenyl tetrazolium bromide)
nm: Nanometer
NGF: Nerve growth factor
NTs: Neuropil threads
NFTs: Neurofibrillary tangles
ROS: Reactive oxygen species
SDS: Sodium dodecyl sulfate
