# Supplementary material for: Phytochemical analysis and anticholinesterase activity of aril of Myristica fragrans Houtt
Source: BMC Chem. 2022 Nov 28;16(1):106. doi: 10.1186/s13065-022-00897-9 (PMC9703800; doi:10.1186/s13065-022-00897-9)
Supplement: Supplementary file 1 — Additional file 1: Table S1. Isolated compounds from the aril of M. fragrans. [file 13065_2022_897_MOESM1_ESM.docx]

**Additional file**

**Compound 1**

**
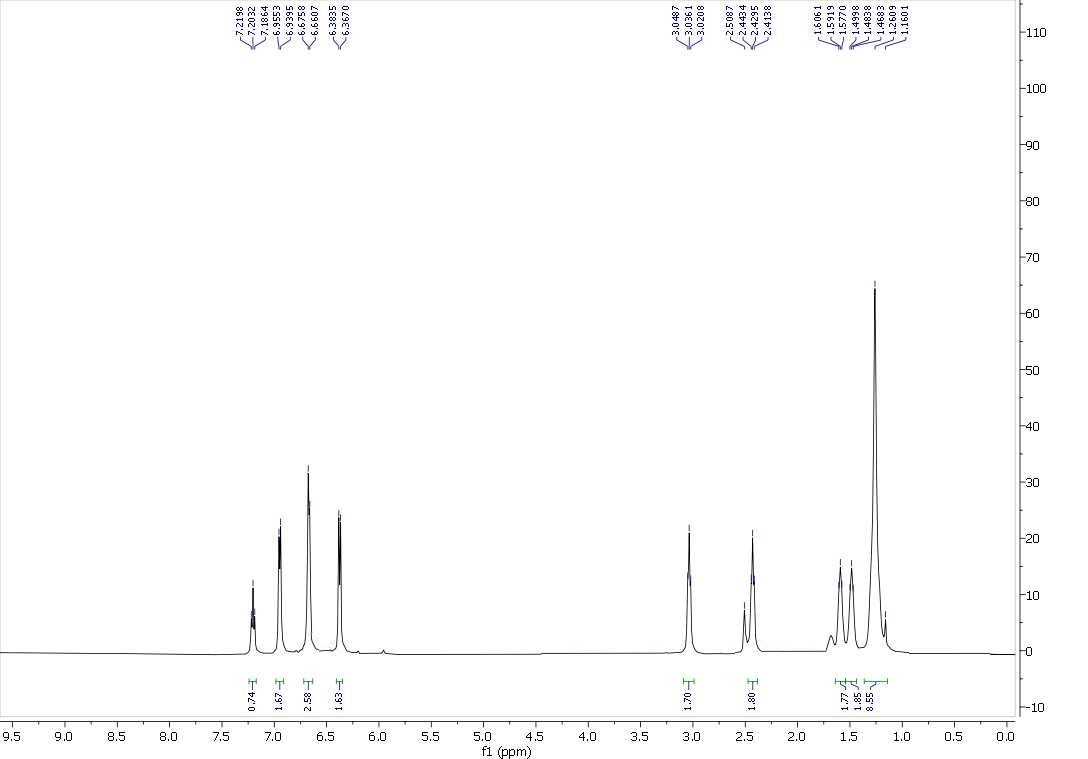
**

**
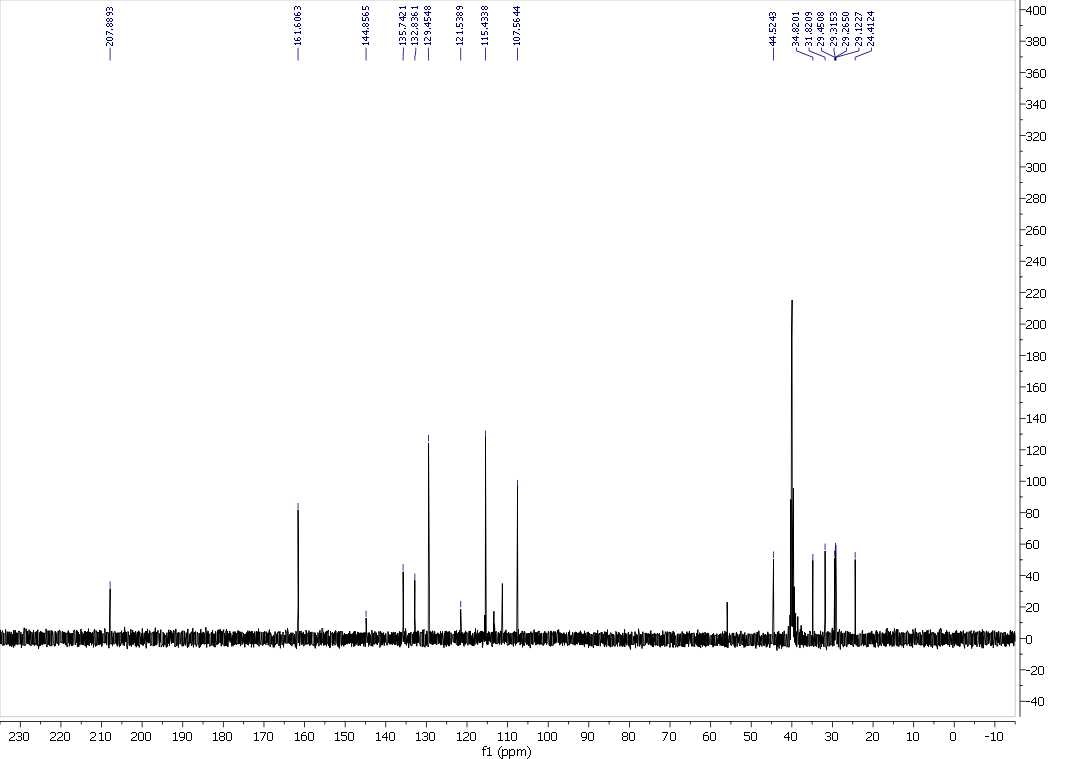
**


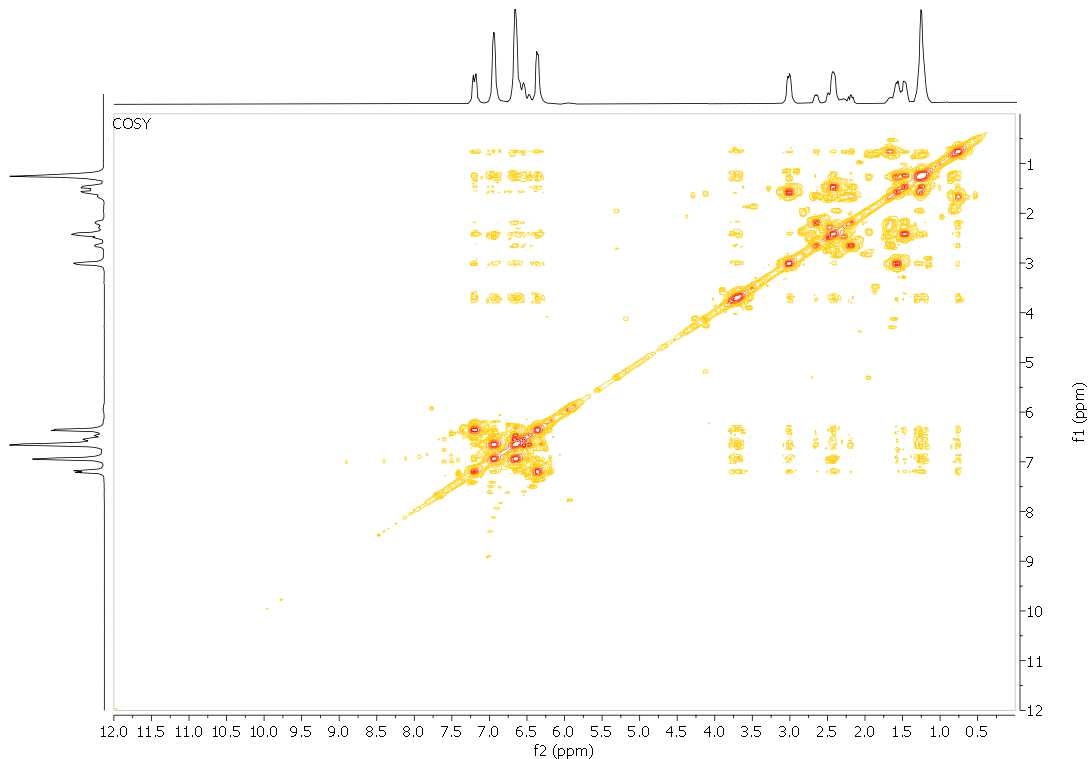


**Compound 2**

**
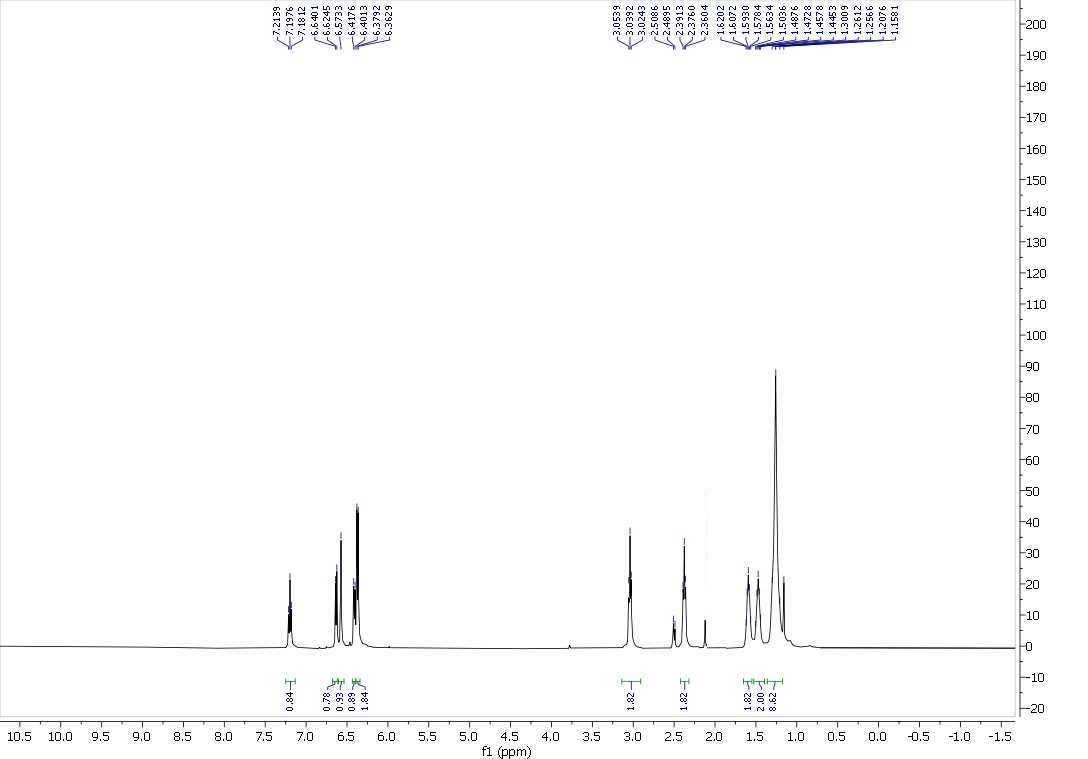
**

**
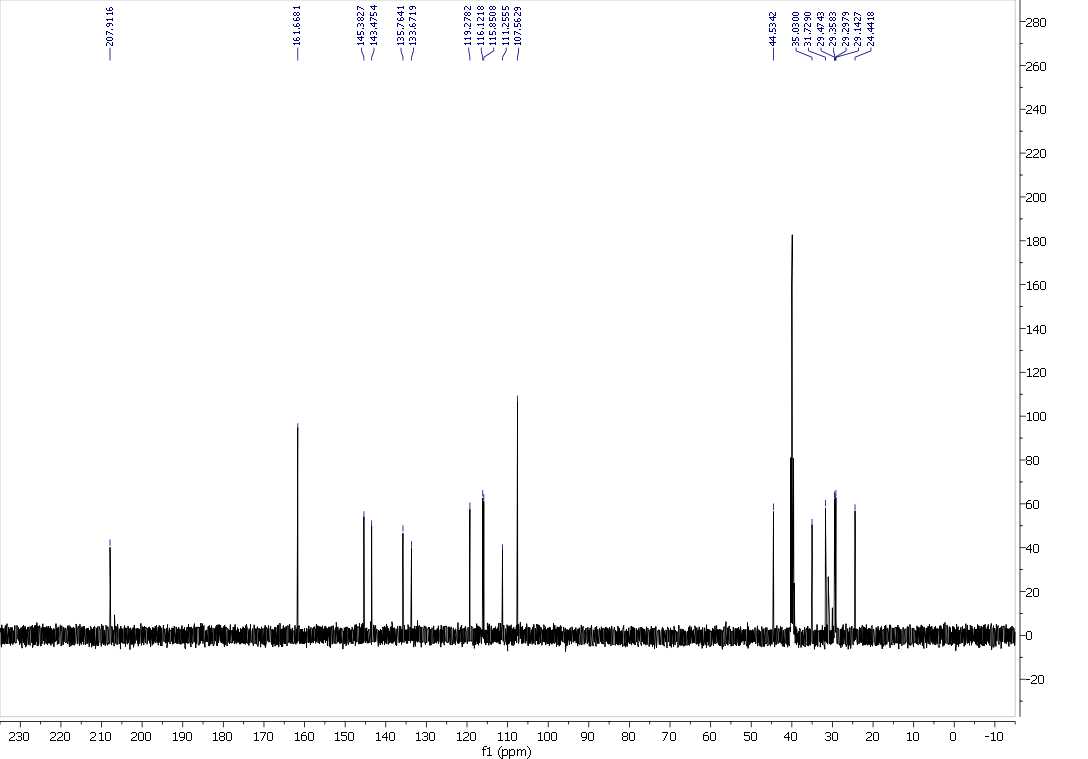
**

**Compound 3**

**
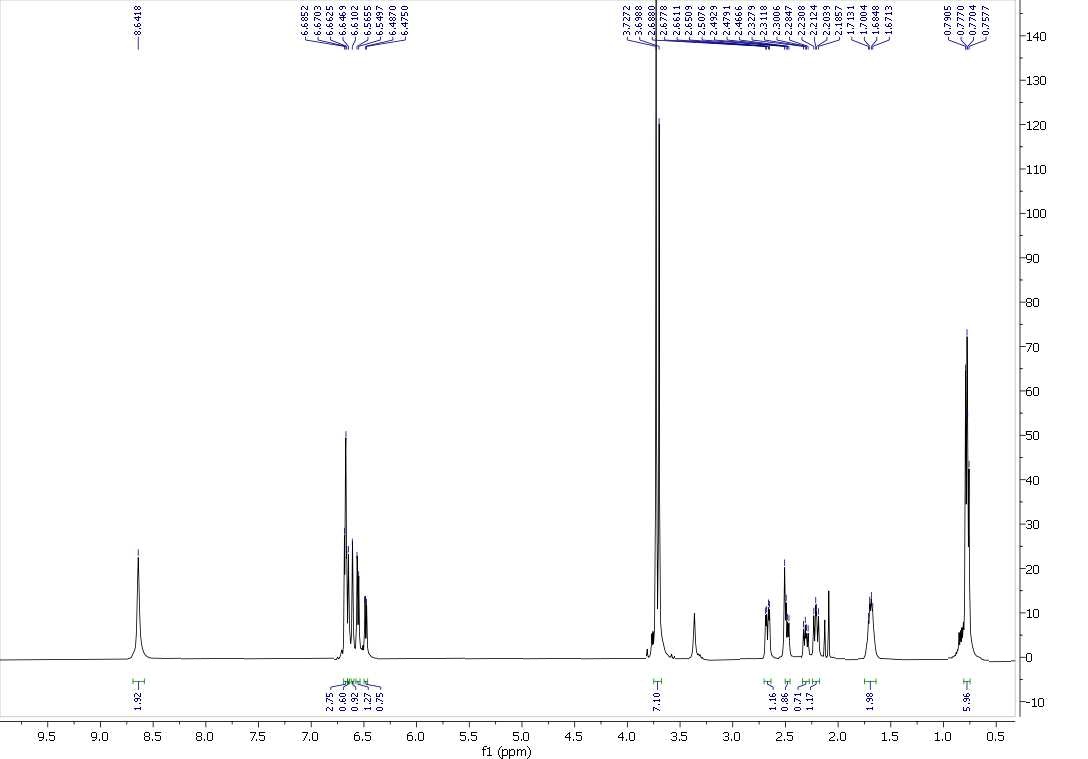
**

**
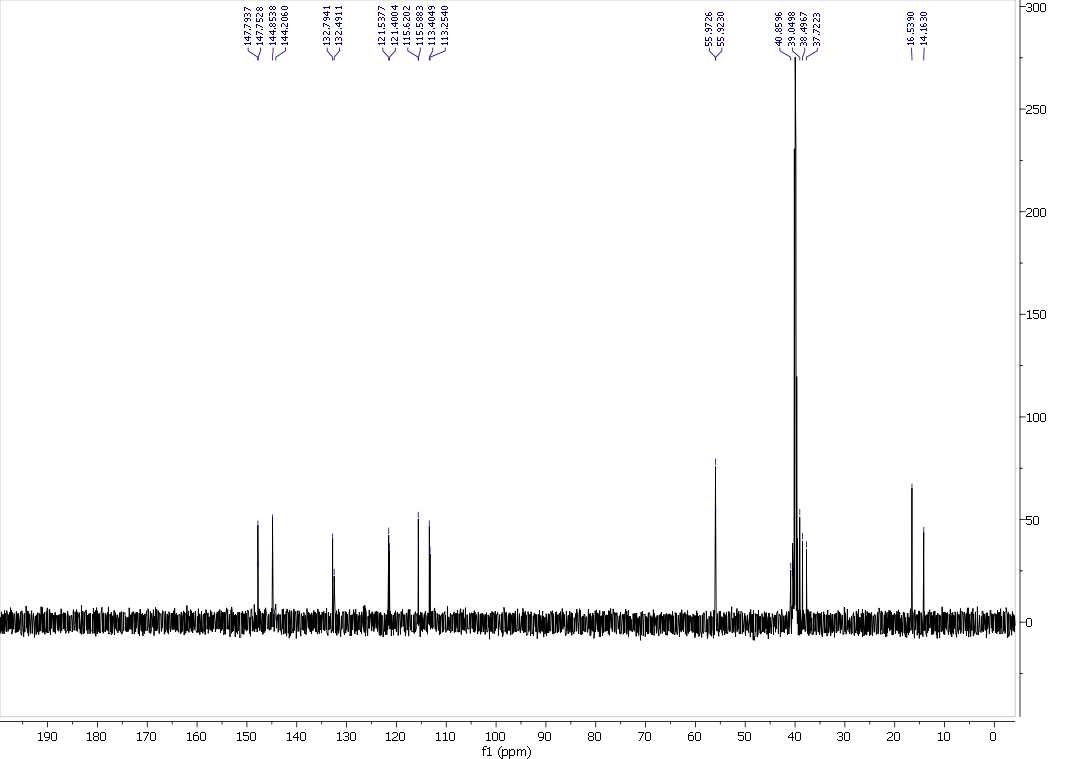
**

**Compound 4**

**
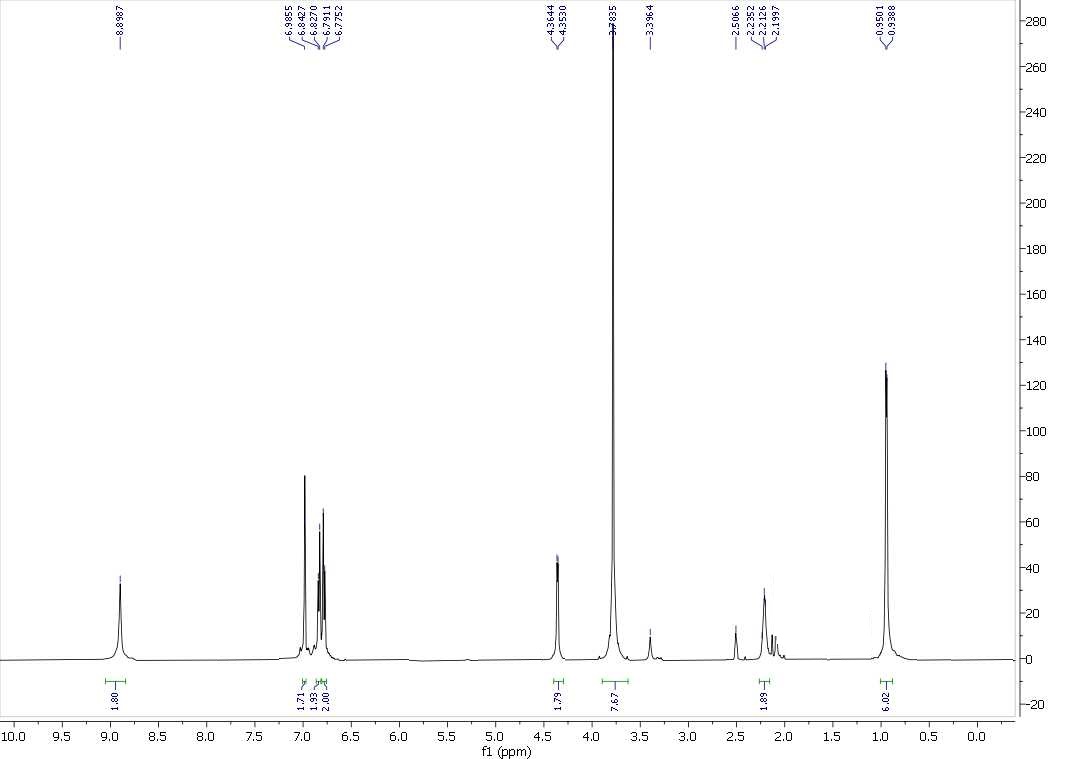
**

**
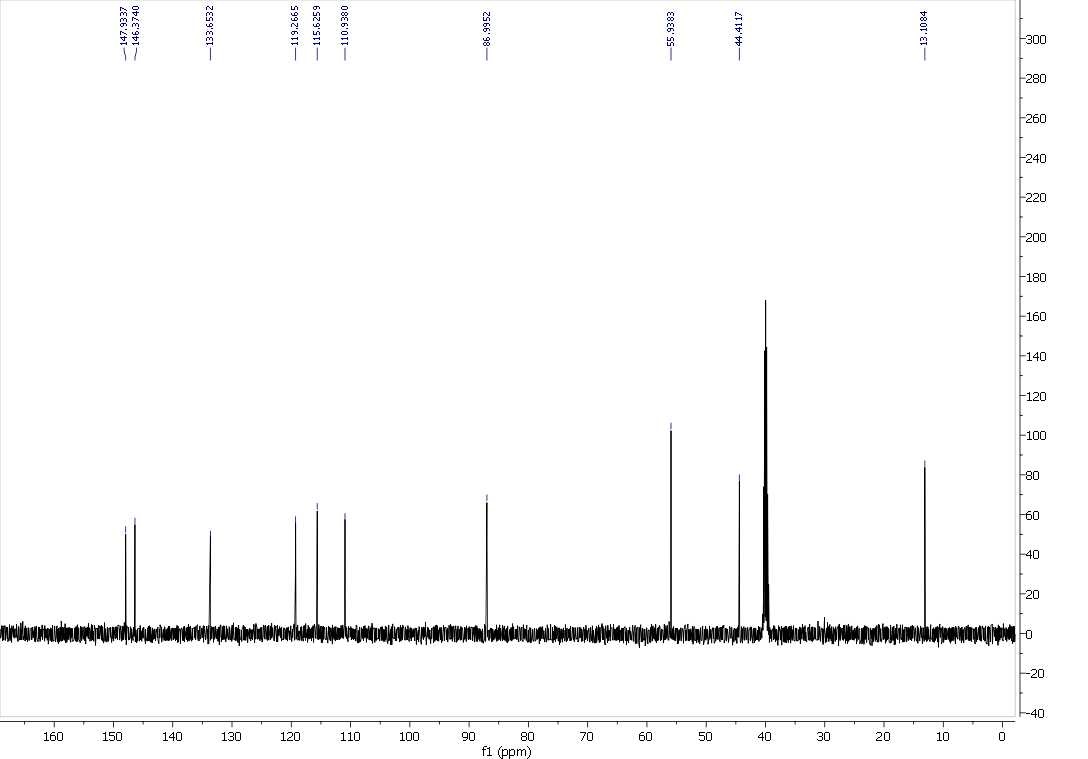
**

**Compound 5**


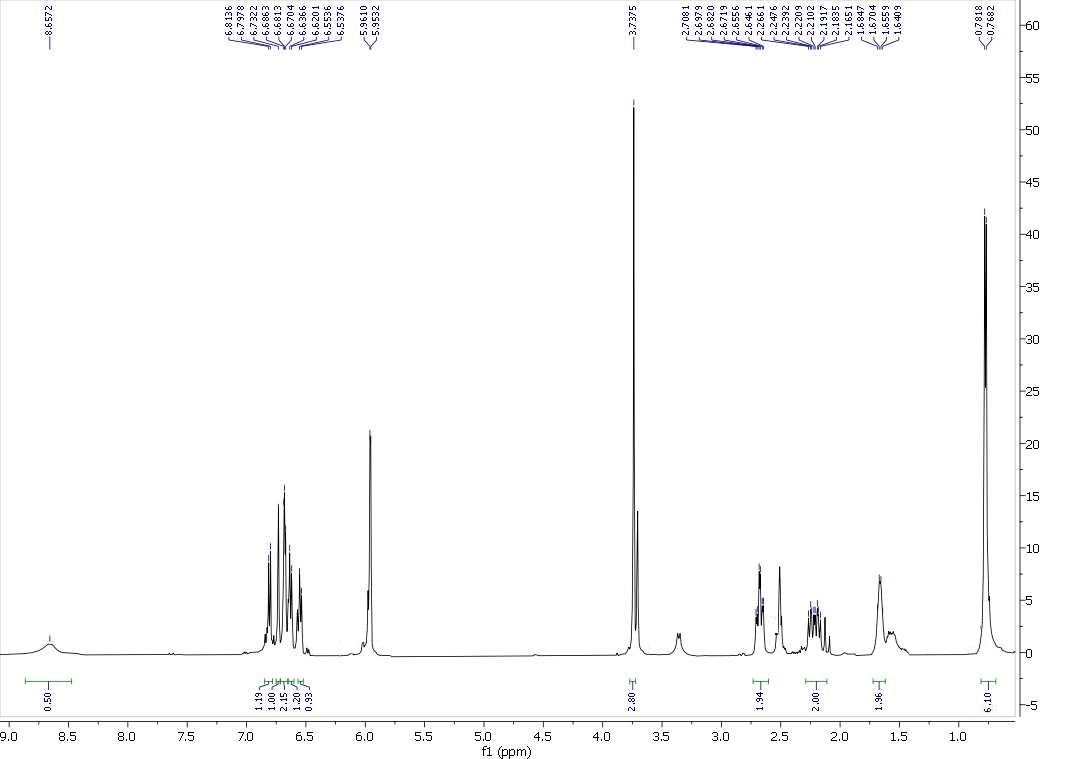


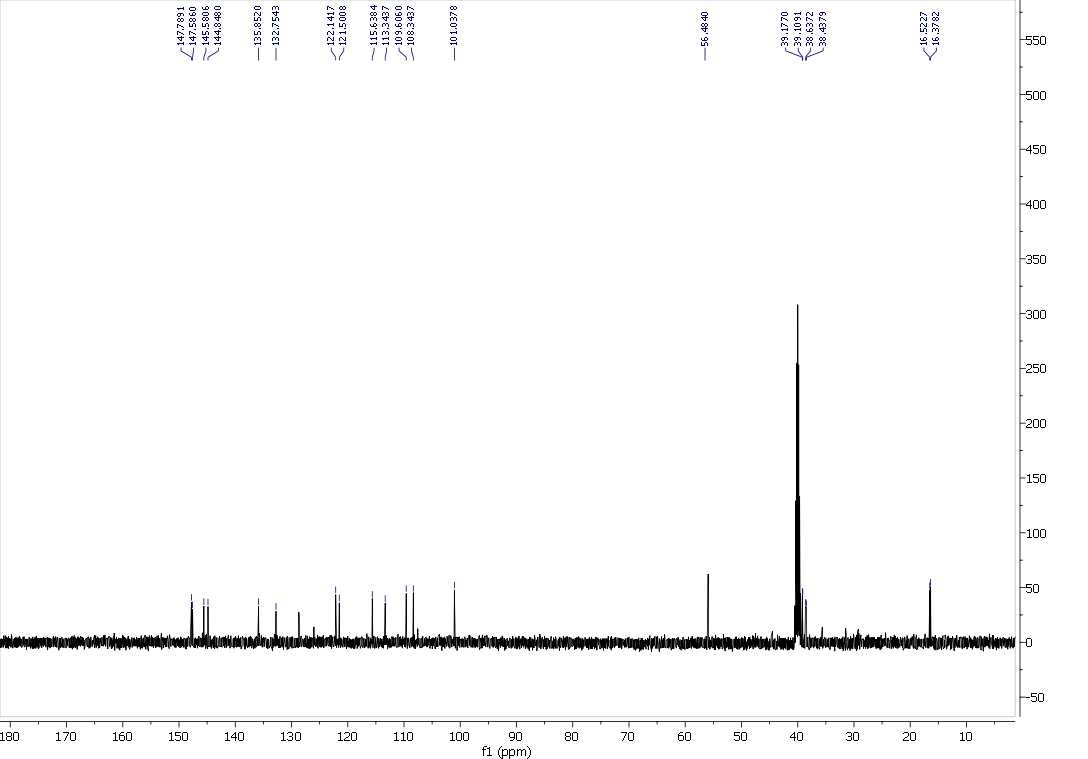


**Compound 6**


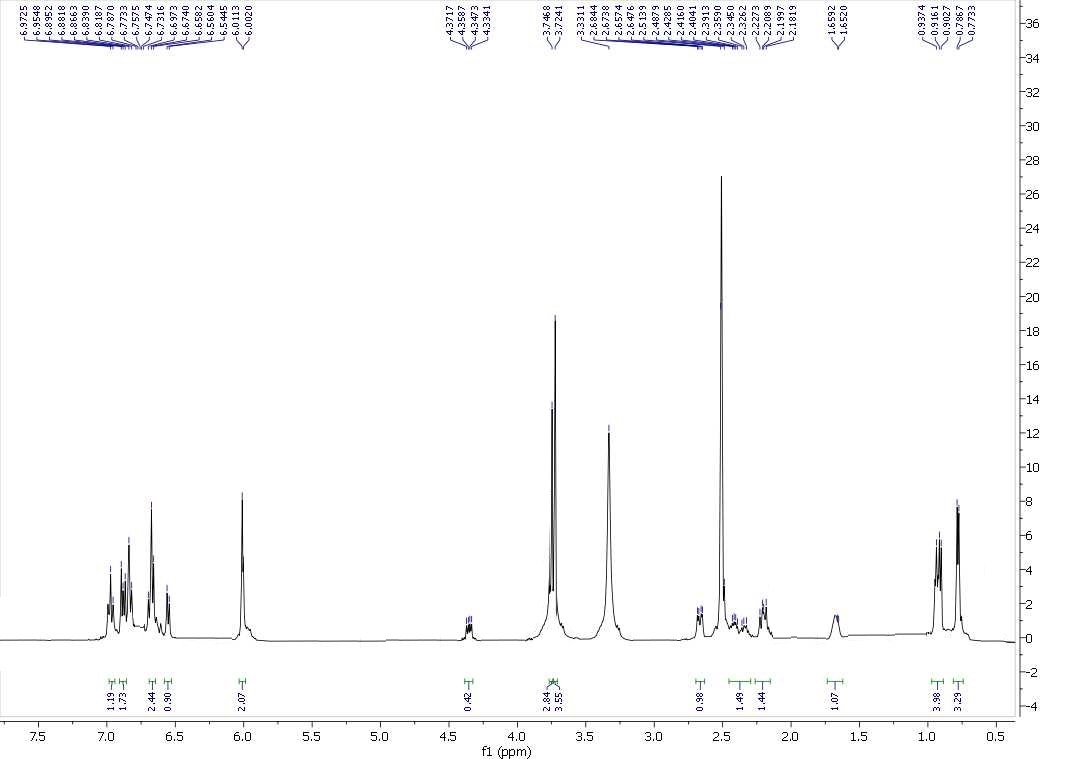


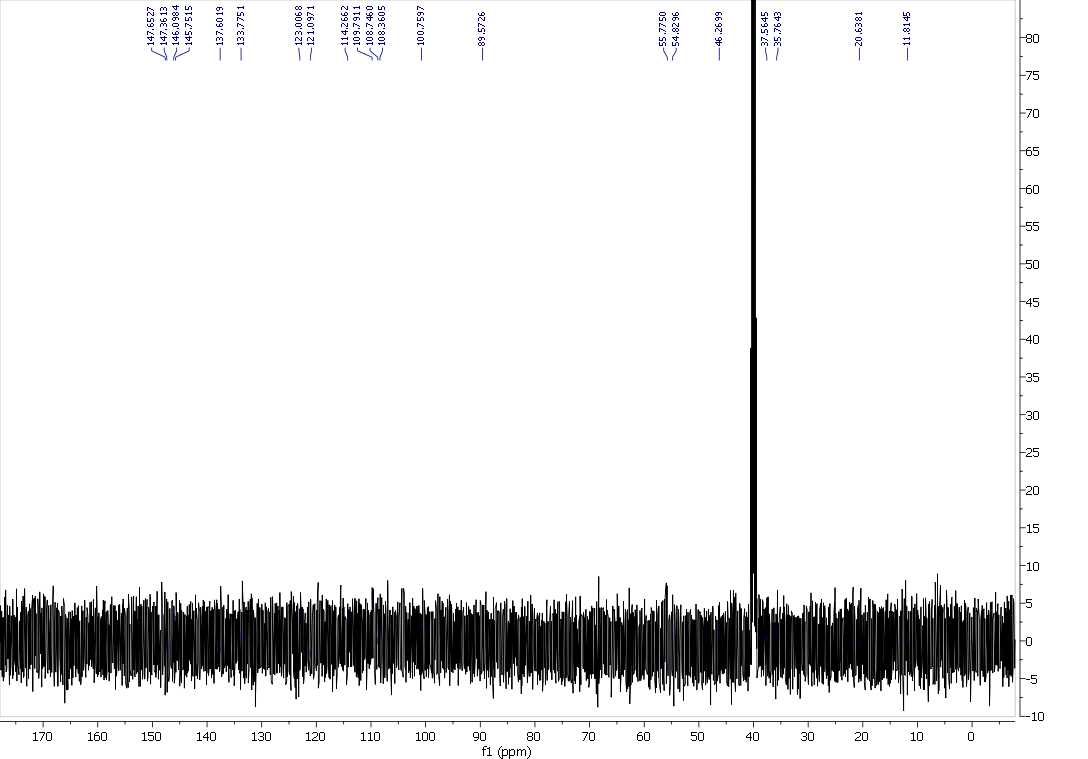


Table S1. Isolated compounds from the aril of *M. fragrans*

| No. | Compound name | No. | Compound name |
| --- | --- | --- | --- |
| 1 | Macelignan [(2R,3S)-1-(3,4 melhylenedioxyphenyl)-2,3-dimethyl-4-(4- hydroxy-3-methoxyphenyl)-butane] | 44 | (+)-Erythro-(1S,2R)-2-(4-allyl-2,6-dimethoxyphenoxy)-1-(3,4,5-trimethoxyphenyl) propan-1-ol |
| 2 | Meso-dihydroguaiaretic acid | 45 | Erythro-2-(4-allyl-2,6-dimethoxyphenoxy)-1-(4-hydroxy-3-methoxyphenyl) propan-1-ol |
| 3 | 2,3-Dimethyl-1,4-bis-(3,4 methylenedioxyphenyl) butan-1-ol | 46 | (+)-Erythro-(1S,2R)-2-(4-allyl-2,6-dimethoxyphenoxy)-1-(4-hydroxy-3-methoxyphenyl) propan-1-ol |
| 4 | Nectandrin B | 47 | Myrislignan [(−)-erythro-(1R,2S)-2-(4-allyl-2,6-dimethoxyphenoxy)-1-(4-hydroxy-3-methoxyphenyl) propan-1-ol] |
| 5 | Fragransin A_2_ | 48 | Threo-2-(4-allyl-2,6-dimethoxyphenoxy)-1-(4-hydroxy-  3-methoxyphenyl) propan-1-ol |
| 6 | Verrucosin [7S,8S,7′R,8′S)-4,4′-dihydroxy-3,3′-dimethoxy-7,7′-epoxylignan] | 49 | (−)-Erythro-(1R,2S)-2-(4-allyl-2,6-dimethoxyphenoxy)-1-(3,4-dimethoxyphenyl) propan-1-ol |
| 7 | Fragransin B_1_ | 50 | Erythro-2-(4-allyl-2,6-dimethoxyphenoxy)-1-(3,4-methylenedioxyphenyl) propan-1-ol acetate |
| 8 | Fragransin B_2_ | 51 | (−)-Erythro-(1R,2S)-2-(4-allyl-2,6-dimethoxyphenoxy)-1-(3,4-methylenedioxyphenyl) propan-1-ol acetate |
| 9 | Fragransin B_3_ | 52 | Erythro-2-(4-allyl-2,6-dimethoxyphenoxy)-1-(3,4-dimethoxyphenyl) propan-1-ol acetate |
| 10 | Fragransin C_1_ | 53 | (1S,2R)-2-(4-Allyl-2,6-dimethoxyphenoxy)-1-(3,4-dimethoxyphenyl) propan-1-ol acetate |
| 11 | Fragransin C_2_ | 54 | Maceneolignan H [(−)-erythro-(1R,2S)-2-(4-allyl-2,6-dimethoxyphenoxy)-1-(3,4-dimethoxyphenyl) propan-1-ol acetate] |
| 12 | Fragransin C_3a_ | 55 | (1S,2R)-2-(4-Allyl-2,6-dimethoxyphenoxy)-1-(4-hydroxy-3-methoxyphenyl) propan-1-ol acetate |
| 13 | Fragransin C_3b_ | 56 | Erythro-2-(4-allyl-2,6-dimethoxyphenoxy)-1-(5-acetoxy-3,4-dimethoxyphenyl) propan-1-ol acetate |
| 14 | Fragransin D_1_ | 57 | 2-(4-Allyl-2,6-dimethoxyphenoxy)-1-(3,4,5-trimethoxyphenyl) propane |
| 15 | Fragransin D_2_ | 58 | (−)-(2R)-2-(4-allyl-2,6-dimethoxyphenoxy)-1-(3,4,5-trimethoxyphenyl)propane |
| 16 | Fragransin D_3_ | 59 | (−)-(2R)-2-(4-allyl-2,6-dimethoxyphenoxy)-1-(3,4-methylenedioxyphenyl) propane |
| 17 | Fragransin E_1_ or machilin F | 60 | Threo-2-(4-allyl-2,6-dimethoxyphenoxy)-1-(4-hydroxy-3-methoxyphenyl) propan-1-ol methyl ether |
| 18 | Austrobailignan-7 | 61 | Erythro-2-(4-allyl-2,6-dimethoxyphenoxy)-1-(4-hydroxy-3-methoxyphenyl) propan-1-ol methyl ether |
| 19 | Licarin A or dehydrodiisoeugenol | 62 | Erythro-2-(4-allyl-2,6-dimethoxyphenoxy)-1-(4-hydroxy-3,5-dimethoxyphenyl) propan-1-ol |
| 20 | (+)-Licarin | 63 | Maceneolignan F [(+)-erythro-(1S,2R)-2-(4-allyl-2,6-dimethoxyphenoxy)-1-(4-hydroxy-3,5-dimethoxyphenyl) propan-1-ol] |
| 21 | (2S,3S)-2,3-Dihydro-2-(4-hydroxy-3,5-dimethoxyphenyl)-7-methoxy-3-methyl-5-(*E*-propenyl) benzofuran | 64 | 2-(4-Allyl-2,6-dimethoxyphenoxy)-1-(4-hydroxy-3-methoxyphenyl) propane |
| 22 | Isolicarin A | 65 | (−)-(2R)-2-(4-Allyl-2,6-dimethoxyphenoxy)-1-(4-hydroxy-3-methoxyphenyl) propane |
| 23 | Odoratisol A [3′-methoxy-licarin A] | 66 | Erythro-2-(4-allyl-2,6-dimethoxyphenoxy)-1-(3-hydroxy-4,5-dimethoxyphenyl) propan-1-ol |
| 24 | Maceneolignan A [(2R,3R)-2,3-Dihydro-2-(4-hydroxy- 3,5-dimethoxyphenyl)-7-methoxy-3-methyl-5 (*E*-propenyl)benzofuran] | 67 | Erythro-2-(4-allyl-2,6-dimethoxyphenoxy)-1-(3,4,5-trimethoxyphenyl) propan-1,3-diol |
| 25 | (2S,3S)-2,3-Dihydro-2-(4-hydroy-3,5-dimethoxyphenyl)- 7-methoxy-3-methyl-5-(*E*-propenyl) benzofuran | 68 | Erythro-2-(4-allyl-2-methoxyphenoxy)-1-(4-hydroxy-3- methoxyphenyl) propan-1-ol |
| 26 | Licarin B | 69 | Threo-2-(4-allyl-2-methoxyphenoxy)-1-(4-hydroxy-3-methoxyphenyl) propan-1-ol |
| 27 | (2S,3S)-2,3-Dihydro-2-(3,4-methylenedioxyphenyl)-7- methoxy-3-methyl-5-(*E*-propenyl) benzofuran | 70 | Erythro-1-(4-hydroxy-3-methoxyphenyl)-2-(2-methoxy-4-(1(*E*)-propenyl) phenoxy) propan-1-ol |
| 28 | 3′-Methoxy-licarin B | 71 | Machilin D [threo-1-(4-hydroxy-3-methoxyphenyl)-2-(2-methoxy-4-(1(*E*)-propenyl) phenoxy) propan-1-ol] |
| 29 | Maceneolignan B [(2R,3R)-3′-methoxy-licarin B] | 72 | Erythro-1-(4-hydroxy-3-methoxyphenyl)-1-methoxy-2-(2-methoxy-4-(1(*E*)-propenyl) phenoxy) propane |
| 30 | (2S,3S)-2,3-Dihydro-2-(5-methoxy-3,4-methylenedioxyphenyl)-7-methoxy-3-methyl-5-(*E*-propenyl) benzofuran | 73 | Threo-1-(4-hydroxy-3-methoxyphenyl)-1-methoxy-2-(2-methoxy-4-(1(*E*)-propenyl) phenoxy) propane |
| 31 | Licarin C | 74 | Threo-1-(4-hydroxy-3,5-dimethoxyphenyl)-2-(2-methoxy- 4-(1(*E*)-propenyl) phenoxy) propan-1-ol |
| 32 | Maceneolignan C | 75 | Myrifralignan C [(1R,2S)-1-(4-hydroxy-3,5-dimethoxyphenyl)-2-(4-(1(*E*)-propenyl)-2-methoxy phenoxy) propan-1-ol] |
| 33 | Maceneolignan D | 76 | Maceneolignan G [(+)-erythro-(1S,2R)-2-(4-acroloyl-2,6-dimethoxyphenoxy)-1-(4-hydroxy-3-methoxyphenyl) propan-1-ol] |
| 34 | Maceneolignan E | 77 | Myristicanol A [erythro-2-(4-(3-hydroxy-1-(*E*)-propenyl)-2,6-dimethoxyphenoxy)-1-(3,4,5-trimethoxyphenyl) propan-1-ol] |
| 35 | (2S,3S)-2-(4-Hydroxy-3-methoxyphenyl)-5-formyl-7-methoxy-3-methyldihydrobenzofuran | 78 | Myristicanol B [erythro-2-(4-(3-hydroxy-1-(*E*)-propenyl)-2,6-dimethoxyphenoxy)-1-(3,4-dimethoxyphenyl) propan-1-ol] |
| 36 | Fragransol A | 79 | Myrislignanometin E [(1R,2S)-2-(4-(3-hydroxy-1-(*E*)-propenyl)-2,6-dimethoxyphenoxy)-1-(4-hydroxy-3-methoxyphenyl) propan-1-ol] |
| 37 | Fragransol B | 80 | Maceneolignan I |
| 38 | Fragransol C | 81 | (−)-Miliusfragranol B |
| 39 | Fragransol D | 82 | Maceneolignan J |
| 40 | Isodihydrocarinatidin | 83 | Maceneolignan K |
| 41 | Dihydrocarinatidin | 84 | 1-Deoxycarinatone [2-[(1S)-2-(4-hydroxy-3-methoxyphenyl)-1-methylethyl]-6-methoxy-4-(prop-2-enyl)phenol] |
| 42 | (+)-Erythro-(1S,2R)-2-(4-allyl-2,6-dimethoxyphenoxy)-1-(3,4-methylenedioxyphenyl) propan-1-ol | 85 | (S)-1-(3,4,5-Trimethoxyphenyl)-2-(3-methoxy-5-(prop-1-yl) phenyl)-propan-1-ol |
| 43 | Erythro-2-(4-allyl-2,6-dimethoxyphenoxy)-1-(3,4,5-trimethoxyphenyl) propan-1-ol |  |  |
